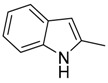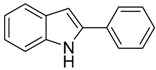# Electrochemical Umpolung of Bromide: Transition-Metal-Free Bromination of Indole C–H Bond

**DOI:** 10.3390/molecules24040696

**Published:** 2019-02-15

**Authors:** Pan Zhang, Jianbin Chen, Wei Gao, Yiting Xiao, Changwei Liu, Shanghui Xu, Xiaoli Yan, Dawei Qin

**Affiliations:** 1 Shandong Provincial Key Laboratory of Molecular Engineering, School of Chemistry and Pharmaceutical Engineering, Qilu University of Technology (Shandong Academy of Sciences), Jinan 250353, China; panzhang11@163.com (P.Z.); gaowei161212@163.com (W.G.); Olivia9802@163.com (Y.X.); ShanghuiXu111@163.com (S.X.); xiaoliyan1a@163.com (X.Y.); qdw109@163.com (D.Q.); 2 Faculty of Science and Forestry, University of Eastern Finland, 80101 Joensuu, Finland; changwl@uef.fi

**Keywords:** umpolung, C–H functionalization, electrochemical organic synthesis, anodic oxidation, sustainable chemistry

## Abstract

A facile and sustainable electrochemical umpolung of bromide ion protocol was developed under mild reaction conditions. Transition metal catalysts and exogenous chemical oxidants were obviated for the bromination of C–H bond. Notably, graphite rod, which is commercially available at supermarkets and is inexpensive, was employed as the electrode material. This operationally easy and environmentally friendly approach accomplished the synthesis of 3-bromoindole in excellent yield and regioselectivity.

## 1. Introduction

**Scheme 1:**
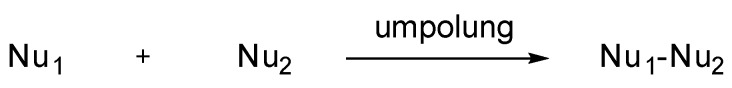
Umpolung concept for coupling two nucleophiles.

Umpolung or polarity inversion, one of the attractive techniques, is a fundamental concept that was first introduced by Seebach and Corey in the 1970s [1,2,3]. It has been widely used in organic synthesis as it can alter the reactivity of a specific functional group for a desirable reaction that would otherwise not be possible [4,5,6,7,8,9,10,11,12,13,14,15,16,17,18,19]. Under the concept, two nucleophiles can couple with each other (Scheme 1). Many functional groups, for instance, cyanides, N-heterocyclic carbenes (NHC), thiamine pyrophosphates (TPP) as well as dithiane moieties, have been treated as the mediators and/or catalysts for umpolung transformation [4,5,6,7,8,9,10,11,12,13,14,15,16,17,18,19].

Halogen ions, especially bromide ions, are typical nucleophiles, which are frequently used for nucleophilic substitution and addition. In order to achieve the umpolung of bromide to bromo-based electrophiles, strong chemical oxidants are regularly required [20,21,22,23,24,25,26,27,28]. However, based on the intrinsic disadvantages of chemical oxidants, such as toxicity, environmental pollution, explosion risk, and economic costs, there is a growing need for green and sustainable approaches for the umpolung of bromides. Electrochemistry represents one of the most sustainable ways because only clean electrons are involved in the process [29,30,31,32,33,34,35,36,37,38,39,40,41,42,43,44,45,46,47,48]. In line with this strategy, in 2015, Bell and Wang reported that bromide ion can be electro-oxidized with observation current and potential oscillations [49]. Xu et al. and Yu et al. described electrochemical oxidation of bromide on platinum electrodes in aqueous acidic solution and ionic liquid, respectively [50,51,52]. Some new materials have also been exploited as electrodes for the electro-oxidation of bromides [53,54]. Inspired by these elegant discoveries, we envisioned that umpolung of bromide can be achieved by electrochemical strategy.

Aromatic bromides are an important class of compounds, which are commonly utilized as the coupling partner in transition-metal-catalyzed cross-coupling reactions [55,56,57,58,59,60,61,62,63,64,65,66,67]. Thus, the development of a synthetic method of arylbromides has attracted tremendous efforts [68,69,70,71,72,73,74,75,76,77,78,79,80,81,82]. Among them, much progress has been made on palladium-catalyzed regioselective bromination of C–H bonds using different agents. Sanford and co-workers elegantly delineated Pd-catalyzed C–H bromination of benzoquinoline with *N*-bromosuccinimide (NBS) as the brominating reagent [83,84,85]. Wan et al. and Jia et al. unraveled that copper halides can be treated as brominating agents in Pd-catalyzed bromination of arenes [86,87]. Notably, stoichiometric chemical oxidants are indispensable in the abovementioned Pd-catalyzed C–H bromination reactions. Moreover, electrophilic bromination of indoles has also been developed with sodium bromide (NaBr) or potassium bromide (KBr) as bromiding agents, which were mediated by strong chemical oxidants [88,89]. Interestingly, NBS was straightforwardly utilized for the electrophilic bromination under ultraviolet UV irradiation [90]. However, the high cost and the toxicity of chemical oxidants is associated with heavy pollution to the environment, making them a major concern for the synthetic industry. In order to overcome the disadvantages inherent in chemical oxidants, Kakiuchi and co-workers elegantly substantiated the Pd-catalyzed bromination of arylpyridine with hydrogen bromide via electrochemical oxidation [91]. Compared with regular palladium-catalyzed C–H bromination, many advances were achieved for this electrochemical protocol. However, the involvement of corrosive acid (HBr) and noble metal as the catalyst was the main drawback. Herein, we report on an electrochemical transition-metal-free C–H bromination with bromide salts as the source under mild conditions with inexpensive electrode materials that are commercially available in supermarkets. [92]

## 2. Results and Discussions

**Figure 1:**
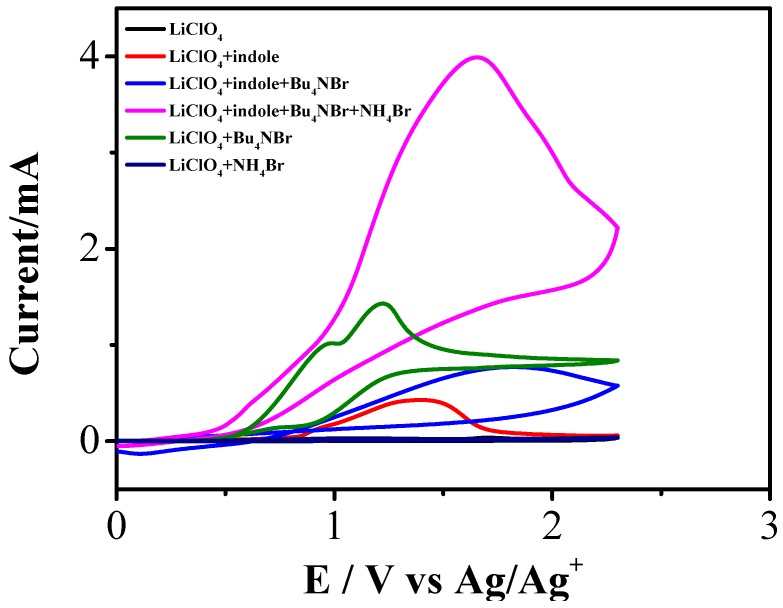
Cyclic voltammetry investigation of indole and bromide salts. Conditions: LiClO_4_ (0.1 M in MeCN) as background supporting electrolyte, glassy carbon as working electrode, Pt wire as counter electrode, Ag in AgNO_3_ (0.01 M)–LiClO_4_ (0.1 M) in MeCN as reference electrode, indole (0.1 M), *n*Bu_4_NBr (0.1 M), and NH_4_Br (0.1 M). The scan rate is 100 mV/s.

To prove the concept, we chose ubiquitous bromide salts, i.e., tetrabutylammonium bromide (*n*Bu_4_NBr) and/or ammonium bromide (NH_4_Br), as the bromide source as well as the supporting electrolyte. In order to gain rational understanding of the oxidative process, cyclic voltammetric (CV) experiments were conducted. As shown in Figure 1 (dark red line), the oxidative peak potential of Br^−^ appeared at 0.96 V and 1.22 V versus Ag/Ag^+^, which were assigned to the corresponding Br·/Br^−^ and Br^+^/Br· redox couple, respectively [5,6,7]. Indole, one of the electron-rich heterocyclics, displayed a profound nucleophilicity, and its CV behavior was surveyed. Notably, the oxidative potential of indole was observed at 1.39 V versus Ag/Ag^+^ (Figure 1, bright red line). Intriguingly, both the peak potentials of Br^−^ and indole unambiguously shifted and diminished to the positive side when equal molar of substrates were added. This scenario can be explained by the hydrogen bonding between the free N–H moiety of indole and the bromide anion (Figure 1, blue line). Interestingly, the addition of equal molar of NH_4_Br to the mixture of *n*Bu_4_NBr and indole resulted in a recovery of the intrinsic peak potential (Figure 1, pink line).

**Figure 2:**
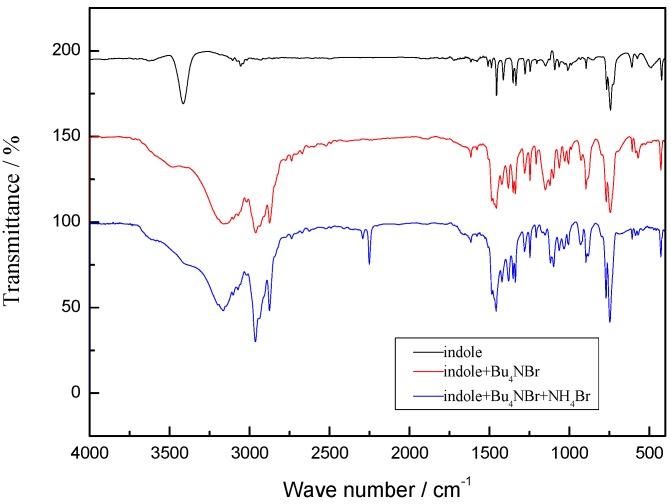
Infrared spectroscopy studies of indole and bromide salts.

With the aim of confirming the existence of hydrogen bonding, infrared (IR) spectroscopy of indole and/or bromide sources was further performed. As shown in Figure 2, the characteristic wavenumber (approximately 3370 cm^−1^) of free indole (free N–H) shifted distinctly to a new broad peak around 3200 cm^−1^ when equal molar of *n*Bu_4_NBr was added to the sample of indole. This phenomenon can again be attributed to the H-bonding interaction between the free N–H and the bromide anion.

With the understanding of electrochemical oxidative course, we carried out the reaction with indole **1** and *n*Bu_4_NBr as well as NH_4_Br to testify the electrochemical umpolung of bromide. Initially, the procedure employed the mixture of *n*Bu_4_NBr and NH_4_Br in order to interrupt the H-bonding with indole and was set up in 1,4-dioxane under a galvanostatic model. Unfortunately, trace of the desired product **2** was formed (Table 1, entry 1). However, when the reaction was run in tetrahydrofuran (THF), 39% of **2** was generated (Table 1, entry 2). Chlorine-containing solvents, such as dichloroethane (DCE), provided moderate efficiency (43% yield, Table 1, entry 3). Then, we screened the aprotic polar media, for instance, dimethylforamide (DMF), dimethyl sulfoxide (DMSO), and ethyl acetate, in which 25% to 58% of the final product was isolated (Table 1, entries 4–6). The ethanol as medium resulted in 20% yield (Table 1, entry 7). Satisfyingly, when the reaction was set up in acetonitrile (MeCN), excellent efficiency was observed (81% yield, Table 1, entry 8). Notably, this transformation was sensitive to water as the yield decreased sharply in the presence of water (Table 1, entry 11). Interestingly, this protocol worked smoothly when the amount of bromide salts was reduced, whereas no significant enhanced performance was detected when the quantity of bromide anion was increased (Table 1, entry 12 vs. 15). It should be noted that this approach almost shut down when only NH_4_Br was applied as the bromide source because of the poor solubility (Table 1, entry 16). In addition, diminished yield was observed with only *n*Bu_4_NBr as the nucleophile (Table 1, entry 17), presumably due to the slowdown in the cathodic reduction of proton to hydrogen. This was the case in the absence of NH_4_Br (Table 1, entries 9–10). Increasing or decreasing the current value and displacement of the graphite rod with platinum plate led to diminished yields.

Next, we focused our attention on the substrate scope. We tested a wide range of indole derivatives, as illustrated in Table 2. Good to excellent conversion of the corresponding starting materials was observed, as indicated by gas chromatography–mass spectrometry (GC–MS) and thin-layer chromatography (TLC). Surprisingly, we observed that most of the 3-bromoindole derivative products were not stable and decomposed quickly at ambient temperature of 28 °C. Furthermore, the titled products could only be stored for a few hours at 4 °C in the refrigerator.

**Scheme 2:**
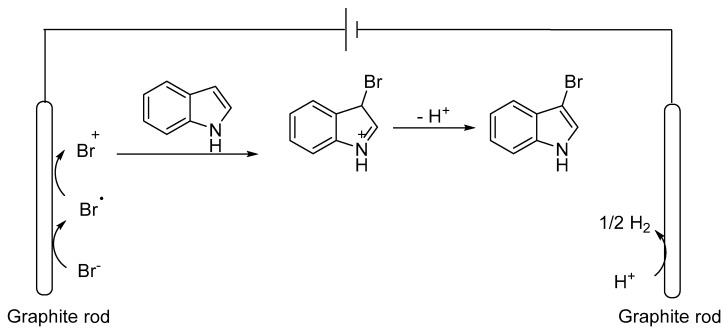
Proposed mechanism for the umpolung coupling of two nucleophiles.

Based on the cyclic voltammetric analysis, a putative mechanism was proposed (Scheme 2). Consecutive anodic oxidation of bromide ion provided bromine cation to furnish the umpolung step. After nucleophilic addition of another nucleophile, indole accomplished the eventual product by releasing a proton. Cathodic evolution of hydrogen via reduction of proton served as the half-reaction.

## 3. Experimental Section

**General Information:** Chemicals and solvents were purchased from commercial suppliers and used as received. ^1^H NMR and ^13^C NMR spectra were recorded on a Bruker AVANCE II 400 (400 MHz) spectrometer (Bruker, Switzerland). Chemical shifts were calibrated using residual undeuterated solvent as an internal reference (CDCl_3_: 7.26 ppm ^1^H NMR, 77.0 ppm ^13^C NMR). The abbreviations used for explaining the multiplicity were as follows: s = singlet, d = doublet, t = triplet, q = quartet, m = multiplet, dd = doublet of doublet, brs = broad singlet. GC–MS spectra were recorded on Agilent 7890B-5977B (Agilent Technologies Inc., California, CA, USA). Both anode and cathode electrodes were carbon rod electrodes (diameter 1.0 cm, length 10 cm). TLC employed 0.25-mm glass silica gel plates. The developed chromatogram was analyzed by a UV lamp (254 nm). Flash chromatography columns were packed with 200–300 mesh silica gel in petroleum (bp: 60–90 °C). Cyclic voltammograms were obtained on a CHI 760E potentiostat. IR spectra (CH Instruments Ins., Shanghai, China) were recorded on a Nicolet10 spectrometer (CH Instruments Ins., Shanghai, China).

**General Procedure for the Electrolysis:** An oven-dried, 10 mL two-neck glass flask was equipped with a magnetic stir bar. The substrate (0.20 mmol, 1 equiv.), *n*Bu_4_NBr (0.20–0.80 mmol), NH_4_Br (0.20–0.40 mmol), and CH_3_CN (10 mL) were combined and added. The flask was equipped with carbon rod electrodes (diameter 1.0 cm, length 10 cm) as both the anode and the cathode. The reaction was initiated at a constant current of 2 mA at room temperature. After complete consumption of the starting material, the solvent was removed with a rotary evaporator. The residue was then subjected to flash column chromatography on silica gel to afford the product.

**Cyclic Voltammetry Studies:** Cyclic voltammetric measurements were carried out in a 50 mL glass vial at room temperature. A glassy carbon disk electrode (diameter is 3.0 mm) was used as the working electrode, while a platinum plate electrode (1.0 × 1.0 cm^2^) was used as counter electrode. The reference Ag/Ag^+^ electrode was made by immersing a sliver wire in a solution of AgNO_3_ (0.01 M)–LiClO_4_ (0.1 M) in MeCN and separated from the reaction by a salt bridge. The scan rate was 100 mV/s.

**Characterization of *3-Bromo-1H-indole*:** Column chromatography was used for purification on a silica gel column using petroleum ether:ethyl acetate = 10:1 as the eluent to give the product as a pale white solid. The data were consistent with the authentic sample. ^1^H NMR (400 MHz, DMSO) **δ**11.41 (s, 1H), 7.48 (d, *J* = 2.7 Hz, 1H), 7.34–7.38 (m, 2H), 7.10–7.14 (m, 1H), 7.03–7.07 (m, 1H). 

## 4. Conclusions

In summary, we developed a concise electrochemical umpolung of bromide ion under mild conditions. The electrolysis utilized cheap electrode material in undivided cell, providing 3-bromoindole in excellent yield.

## Figures and Tables

**Table 1:**
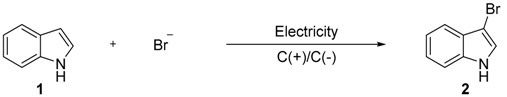
Reaction optimization. ^a.^

Entry	Bromide Source	Solvent	Constant Current	Yield (%)
1	*n*Bu_4_NBr/NH_4_Br (2 equiv.-2 equiv.)	1,4-dioxane	2 mA	<5
2	*n*Bu_4_NBr/NH_4_Br (2 equiv.-2 equiv.)	THF	2 mA	39
3	*n*Bu_4_NBr/NH_4_Br (2 equiv.-2 equiv.)	DCE	2 mA	43
4	*n*Bu_4_NBr/NH_4_Br (2 equiv.-2 equiv.)	DMF	2 mA	25
5	*n*Bu_4_NBr/NH_4_Br (2 equiv.-2 equiv.)	DMSO	2 mA	54
6	*n*Bu_4_NBr/NH_4_Br (2 equiv.-2 equiv.)	EtOAc	2 mA	58
7	*n*Bu_4_NBr/NH_4_Br (2 equiv.-2 equiv.)	EtOH	2 mA	20
8	*n*Bu_4_NBr/NH_4_Br (2 equiv.-2 equiv.)	MeCN	2 mA	81
9	*n*Bu_4_NBr/NaBr (2 equiv.-2 equiv.)	MeCN	2 mA	60
10	*n*Bu_4_NBr/KBr (2 equiv.-2 equiv.)	MeCN	2 mA	28
11	*n*Bu_4_NBr/NH_4_Br (2 equiv.-2 equiv.)	MeCN/H_2_O (10-2 mL)	2 mA	12
12	*n*Bu_4_NBr/NH_4_Br (1 equiv.-1 equiv.)	MeCN	2 mA	80
13	*n*Bu_4_NBr/NH_4_Br (1 equiv.-1 equiv.)	MeCN	2 mA	62 ^b^
14	*n*Bu_4_NBr/NH_4_Br/NaBr (1 -0.5-0.5 equiv.)	MeCN	2 mA	64
15	*n*Bu_4_NBr/NH_4_Br (4 equiv.-2 equiv.)	MeCN	2 mA	82
16	NH_4_Br (2 equiv.)	MeCN	2 mA	<5
17	*n*Bu_4_NBr (2 equiv.)	MeCN	2 mA	15

^a^ Reaction conditions: graphite rod anode and graphite rod cathode, indole 1 (0.2 mmol), bromide salts as indicated, solvents (10 mL), 2 mA, air, 11.5 h, room temperature. ^b^ under O_2_.

**Table 2:** Testing of indole derivatives.

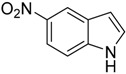	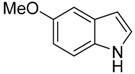	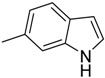	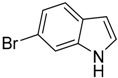
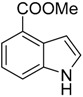	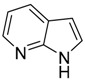